# Effect of supplementing lysins and methionine to low-protein diets on growth performance, hepatic antioxidant capacity, immune status, and glycolytic activity of tibetan sheep

**DOI:** 10.1186/s12864-024-10480-2

**Published:** 2024-06-04

**Authors:** Qiurong Ji, Fengshuo Zhang, Quyangangmao Su, Tingli He, Zhenling Wu, Kaina Zhu, Xuan Chen, Zhiyou Wang, Shengzhen Hou, Linsheng Gui

**Affiliations:** Qinghai University College of Agriculture and Animal Husbandry, Xining, 810016 China

**Keywords:** Amino acid, Tibetan lamb, Transcriptomic analysis, Differentially expressed genes

## Abstract

Reducing the levels of dietary protein is an effective nutritional approach in lowering feed cost and nitrogen emissions in ruminants. The purpose of this study was to evaluate the effects of dietary Lys/Met ratio in a low protein diet (10%, dry matter basis) on the growth performance and hepatic function (antioxidant capacity, immune status, and glycolytic activity) in Tibetan lambs. Ninety two-month-old rams with an average weight of 15.37 ± 0.92 kg were randomly assigned to LP-L (dietary Lys/Met = 1:1), LP-M (dietary Lys/Met = 2:1) and LP-H (dietary Lys/Met = 3:1) treatments. The trial was conducted over 100 d, including 10 d of adaption to the diets. Hepatic phenotypes, antioxidant capacity, immune status, glycolytic activity and gene expression profiling was detected after the conclusion of the feeding trials. The results showed that the body weight was higher in the LP-L group when compared to those on the LP-M group (*P* < 0.05). In addition, the activities of the catalase (CAT) and glutathione peroxidase (GSH-Px) in the LP-L group were significantly increased compared with the LP-M group (*P* < 0.05), while the malondialdehyde (MDA) levels in LP-H group were significantly decreased (*P* < 0.05). Compared with LP-H group, both hepatic glycogen (*P* < 0.01) and lactate dehydrogenase (LDH) (*P* < 0.05) were significantly elevated in LP-L group. For the LP-L group, the hepatocytes were arranged radially with the central vein in the center, and hepatic plates exhibited tight arrangement. Transcriptome analysis identified 29, 179, and 129 differentially expressed genes (DEGs) between the LP-M vs. LP-L, LP-H vs. LP-M, and LP-H vs. LP-L groups, respectively (Q-values < 0.05 and |log2Fold Change| > 1). Gene Ontology (GO) and correlation analyses showed that in the LP-L group, core genes (*C1QA* and *JUNB*) enriched in oxidoreductase activity were positively correlated with antioxidant indicators, while the *MYO9A* core gene enriched in the immune response was positively associated with immune indicators, and core genes enriched in molecular function (*PDK3* and *PDP2*) were positively correlated with glycolysis indicators. In summary, low-protein diet with a low Lys/Met ratio (1:1) could reduce the hepatic oxidative stress and improve the glycolytic activity by regulating the expression of related genes of Tibetan sheep.

## Introduction

The Tibetan sheep (*Ovis aries*) mainly distributed on the Qinghai-Tibetan Plateau at altitudes of over 3000 m [1], which provide a wide variety of resources including meat, milk, fuel, and pelage for local herdsmen in a region characterized by extreme cold and low oxygen levels [2]. Currently, normal dietary crude protein (CP) concentration is about 14–16% in the Tibetan sheep production. Lowering the dietary protein levels can enhance nitrogen (N) utilization and reduce the excretion of N into the environment, as well as lessening seasonally feed costs [3]. Previous studies have shown that supplementing dietary lysins (Lys) and methionine (Met) to low-protein diet may balance the fat accretion and improve protein synthesis [4]. Therefore, we are interested in whether the impacts of low-protein diets on hepatic function are associated with dietary proportions of Lys and Met.

The liver is the most intricate metabolic organ involved in a variety of physiological functions. Via removing harmful metabolites and exogenous microorganisms, the liver was responsible for the organismal immune balance [5]. Additionally, the liver synthesized several enzymes (e.g., glutamic-pyruvic transaminase, glutamic oxalacetic transaminase, and cholinesterase) and then participated in glycogen storage, bile secretion, and protein synthesis [6]. Previously, supplementation of the dietary isoleucine to low-protein diet increased the enzyme activity of acyl-CoA oxidase 1 (ACOX1) in the liver, thereby reducing the fat deposition of broilers [7]. Thus, comprehensive understandings of the physiological function of the liver contribute to improving health status and production in livestock.

Lys is an exogenous essential amino acid, absorbed by the digestive tract epithelial cells and directed to tissues and organs where it acts in the synthesis and deposition of protein [8]. Lys served as a precursor of carnitine and was be involved in collagen production, thereby affecting the hepatic histological structure [9]. It is well established that Met is the first-limiting amino acids for ruminants, depending on dietary composition. It was involved in various physiological processes, such as DNA methylation, protein synthesis, and antioxidant balance [10]. Previous studies conducted in dairy cow observed that supply of Met altered the synthesis of low-density lipoproteins (VLDL), thereby inhibiting the accumulation of triacylglycerol in the liver [11]. Thus, to maintain a protein-amino acid balance, essential amino acids should be supplemented when decreasing the levels of dietary protein while diminishing dietary protein [12].

To date, there are no available reports on the influence of different proportions of amino acids on Tibetan sheep under low protein diet. Therefore, the objective of our experiment was to determine the effect of supplementing Lys to Met in a low-protein diet on the growth performance, antioxidant capacity, immune function, and glycolytic activity in the liver of Tibetan sheep.

## Materials and methods

### Ethical statement

All animal protocols were approved by the Committee of Experimental Animal Care and Handling of the Animal Care Committee of Qinghai University, China.

### Animal diets and sample collection

A total of ninety two-month-old weaned non-neutered male lambs with an average body weight of 15.37 ± 0.92 kg were randomly divided them into 3 different treatments that were fed diets with Lys: Met ratios of 1:1 (LP-L group), 2:1 (LP-M group), and 3:1 (LP-H group). This study was carried out between April and July 2022 at the Jinzang sheep farm in Haiyan Country, Qinghai Province, China. The total experimental period was 100 d, including 10 d of acclimatization and 90 d of actual periods. Throughout the experiment, lambs were fed a 10% crude protein (CP) diet with 70% concentration and 30% forage on a dry matter basis. Fresh drinking water and feed were provided *ad* libitum. The ingredients and chemical composition of the diet are presented in Table 1. The crude protein and crude fat were determined the standard protocol of the Association of Official Analytical Chemists (AOAC) [13]. Neutral detergent fiber and acid detergent fiber was analyzed as described following the methods of Van Soest et al. (1991) [14]. Commercial amino acids were purchased from Henan Bang Lai Industrial Co., LTD (Zhengzhou, China). The digestible energy was calculated. After the end of experimental period, nine lambs (*n* = 3 per treatment) were selected and slaughtered according to the animal welfare procedures. Samples of liver were collected under aseptical condition. Part of each tissue sample was fixed in 4% paraformaldehyde for histological examination, while the remainder was snap-frozen in liquid nitrogen and stored in a -80℃ freezer for RNA extraction.

**Table 1:** Dietary concentrate composition and nutrient levels (% of DM)

Items	LP-L	LP-M	LP-H
Ingredient (%)			
Oat hay	15.000	15.000	15.000
Oat silage	15.000	15.000	15.000
Corn	36.533	37.100	37.100
Wheat	7.700	7.700	7.700
Soybean meal	0.700	0.700	0.700
Rapeseed meal	7.000	7.000	7.000
Cottonseed meal	0.700	0.700	0.700
Maize germ meal	0.700	0.700	0.700
Palm meal	11.200	11.200	11.200
NaCl	0.350	0.350	0.602
Limestone	0.350	0.441	0.700
Baking soda	0.070	0	0.070
Premix ^1)^	2.940	2.940	2.940
Lys	1.386	0.931	0.483
Met	0.371	0.238	0.105
Total	100.000	100.000	100.000
Nutrient levels			
DE (MJ·kg^− 1^) ^2)^	10.760	10.840	10.840
Crude protein	9.940	9.980	9.980
Ether extract	2.850	2.870	2.870
Crude fiber	22.470	22.61	22.610
Neutral detergent fiber	33.720	33.77	33.770
Acid detergent fiber	23.370	23.39	23.390
Ca	0.421	0.424	0.424
P	0.171	0.172	0.172

The premix provided the following per kg of diets: Cu 9.0 mg, Fe 33 mg, Zn 15 mg, Mn 24 mg, Se 0.24 mg, I 0.30 mg, Co 0.12 mg, VA 4 000 IU, VD 2 400 IU, VE 1 500 IU; ^2)^Digestible energy is a calculated value, whereas the others are measured values

### Growth performance

The body weight of Tibetan sheep were recorded before the morning feeding at the beginning and end of the trial. The fattening period was 90 d, during which average daily gain was calculated.

### Enzyme linked immunosorbent assay (ELISA)

Approximately 1.0 g of liver sample was homogenized in ice-cold physiological saline at a ratio of 1:9 (w/v) and subsequently centrifuged at 3500×g at 4 ^о^C for 15 min. The antioxidant capacity (catalase, superoxide dismutase, total antioxidant capacity, glutathione peroxidase and malondialdehyde), immune function (IgA, IgG and IgM), and glycolytic activity (hepatic glycogen, lactate dehydrogenase, malate dehydrogenase and succinate dehydrogenase) of supernate were dealt with the ELISA kits specific to sheep (Nanjing Jiancheng Bioengineering Institute, Nanjing, China) according to the manufacturer’s protocol. The enzymatic activity was analyzed by the microplate reader (Multiskan™ FC, Thermo Fisher Scientific, CA, USA).

### Histological analysis

The fixed tissues were embedded in paraffin and cut into 5-µm sections using a rotary microtome (HS3345, Haifuda Technology,. Ltd, Beijing, China). The tissue sections were stained with hematoxylin and eosin, and then examined by the digital microscope (DP2-BSW, Olympus Corporation, Tokyo, Japan) at 20×magnification. Cross-sectional areas were measured using Image-Pro Plus 6.0 software (Media Cybernetics Inc., Bethesda, MD, USA).

### RNA isolation, library preparation, and sequencing

Total RNA was isolated from 9 liver samples (*n* = 3 per treatment) using TRIzol reagent (#15,596,018, Thermo Fisher Scientific, CA, USA). mRNA was purified from the total RNA using Oligo (dT) (Thermo Fisher, CA, USA) magnetic beads and was fragmented using ABclonal First Strand Synthesis Reaction Buffer. After total RNA was extracted, eukaryotic mRNA was enriched by Oligo (dT) beads. Then the enriched mRNA was fragmented into short fragments using fragmentation buffer and reversly transcribed into cDNA by using NEB Next Ultra RNA Library Prep Kit for Illumina (NEB #7530, New England Biolabs, Ipswich, MA, USA). The purified double-stranded cDNA fragments were end repaired, A base added, and ligated to Illumina sequencing adapters. The ligation reaction was purified with the AMPure XP Beads (1.0X). And polymerase chain reaction (PCR) amplified. The resulting cDNA library was sequenced using Illumina Novaseq6000 by Gene Denovo Biotechnology Co. (Guangzhou, China).

### Quality control and read mapping

The raw reads were translated into sequence reads using CASAVA (Version 1.8.2) and expressed in the FASTQ format. To obtain clean reads, the SOAPnuke package (Version 2.X) was used to trim low-quality reads, sequence connectors, and reads with uncertain N content. The clean reads were then sequenced and aligned with the reference genome (*Ovis aries* version 4.0 https://www.ncbi.nlm.nih.gov/genome/?term=Ovis+aries) using HISAT2 online software (http://daehwankimlab.github.io/hisat).

### Differentially expressed genes (DEGs) analysis

Genes showing differential expression between different samples were identified using DESeq2 with the criteria Q-value < 0.05 and |log2Fold Change| > 1 [15].

### Gene Ontology (GO) enrichment, protein-protein interaction (PPI) network and correlation analysis

To further explore the potential functions of the DEGs, GO enrichment analysis was performed using clusterProfiler (Version 4.0). PPI networks of the DEGs were constructed using the STRING database and visualized using the Cystoscape plug-in cytoHubba (Version 3.9.1) and the maximal clique centrality algorithm to identify hub genes.

OmicShare tools (Version 3.0) were used to conduct correlation network analysis. The data used for the correlation analysis were from fluorescence quantitative measurements and ELISA results. Nine samples (*n* = 3 per treatment) were selected and carried out the correlation analysis.

### Verification of RNA-Seq data with quantitative reserve

Six DEGs were selected from the three groups for verification using RT-qPCR. glyceraldehyde-3-phosphate dehydrogenase (GAPDH) was used as an internal reference **(**Table 2**).** The primers were synthesized by Biotechnology (Shanghai) Co., Ltd (China). The PCR used the RNA from step 2.5 as a template and the reaction system included 10 µL of Universal SYBR Green qPCR Mix, 1 µL of each upstream and downstream primer, and 2 µL of template, made up to 20 µL with nuclease-free water The qPCR amplification program consisted of 40 cycles at 95 ℃ for 30 s, 95 ℃ for 10 s, and 60 ℃ for 30 s. The 2^− ΔΔ Ct^ method was used to calculate the relative gene expression. Nine samples (*n* = 3 per treatment) were selected and carried out the correlation analysis.

**Table 2:** Primers used in qRT-PCR

Name	GenBank accession	Primer sequence (5’-3’)	Tm (℃)	Product length
GAPDH	XM_060411593.1	F-GACCTGCCGCCTGGAGAAAC	60℃	120 bp
		R-AGAGTGAGTGTCGCTGTTGAAGTC		
C1QA	XM_004005142.5	F-AAGGAGAAGAAGTGGGAAGGGATG	60℃	124 bp
		R-TTCCTGCGTCCAATCTTCAACAC		
C1QB	XM_042244592.1	F-AAGGGTGAATCGGGAGACTACAG	60℃	125 bp
		R-TCGTTGGTGTTGGTGATAATGTGG		
C1QC	XM_042244594.1	F-CTGGTCAAGTTCAACGAGGTCATC	60℃	107 bp
		R-GTGTGGAAGACGAAGTAGTAGAAGC		
CFD	XM_012178435.3	F-TCCTCCTCGGGACAGCCTTG	60℃	138 bp
		R-CTATCAGGAAGCCTCCGCACAC		
LAPTM5	XM_004005050.5	F-CCAGCCAGGACGGTATGACTC	60℃	77 bp
		R-GAGGACAGTGATGAAGGCAATGG		
PSAP	XM_060406597.1	F-GTGCTCCGCTCTCAACCTCTG	60℃	96 bp
		R-GCCACCACCTCCGCCATATC		

Glyceraldehyde-3-phosphate dehydrogenase: GAPDH. Complement C1q A chain: C1QA. Complement C1q B chain: C1QB. Complement C1q C chain: C1QC. Complement factor D: CFD. Lysosomal protein transmembrane 5: LAPTM5 Prosaposin: PSAP

## Results

### Growth performance

Although the difference was not significant (Table 3), the average daily gain increased with decrease in dietary Lys/Met ratio (*P* > 0.05). Additionally, the final body weight was higher in the LP-L group when compared to those on the LP-M group (*P* < 0.05).

**Table 3:** Growth performance of Tibetan sheep supplemented with different Lys/Met ratio

Item	LP-L	LP-M	LP-H	*P*-value
Initial weight (kg)	15.79 ± 0.16	15.54 ± 0.17	15.47 ± 0.19	0.393
Final body weight (kg)	37.39 ± 0.37^a^	36.19 ± 0.32^b^	36.45 ± 0.37^ab^	0.049
Average daily weight gain (g/d)	240.13 ± 4.34	229.20 ± 3.87	231.11 ± 3.51	0.122

### Antioxidant, immune response, and glycolytic enzyme activity indices

As shown in Table 4, the catalase (CAT) and glutathione peroxidase (GSH-Px) activities in LP-L group demonstrated significantly increased compared to LP-M group (*P* < 0.05), while the malondialdehyde (MDA) levels in LP-L group were significantly decreased than those in LP-H group (*P* < 0.05). In addition, the indicators of hepatic glycogen and lactate dehydrogenase (LDH) in LP-L group were significantly increased compared with LP-M group (*P* < 0.05).

**Table 4:** Antioxidant, immune response index and sugar degrading enzyme activity index assays

Item	LP-L	LP-M	LP-H	*P*-value
Oxidation indexes				
Catalase (CAT)	123.1 ± 1.95^a^	78.48 ± 9.05^b^	94.70 ± 11.94^ab^	0.036
Glutathione peroxidase (GSH-Px)	1134.02 ± 95.71^a^	982.12 ± 35.67^b^	1023.41 ± 12.65^ab^	0.075
Malondialdehyde (MDA)	5.34 ± 0.34^b^	6.16 ± 0.19^b^	7.55 ± 0.37^a^	0.001
Superoxide dismutase (SOD)	470.40 ± 16.16	477.49 ± 36.57	434.33 ± 10.14	0.411
Total antioxidant capacity (T-AOC)	23.66 ± 1.63	20.45 ± 2.00	20.07 ± 1.29	0.316
Immune indices				
Immunoglobulin A (IgA)	270.77 ± 10.06	255.65 ± 1.60	265.52 ± 8.32	0.415
Immunoglobulin G (IgG)	842.87 ± 86.07	832.69 ± 62.55	755.83 ± 4.24	0.579
Immunoglobulin M (IgM)	1415.76 ± 105.38	1414.24 ± 31.64	1438.48 ± 61.56	0.966
Glycolytic enzyme activity indexes				
Hepatic glycogen	22.94 ± 0.55^a^	14.93 ± 0.73^c^	17.07 ± 0.28^b^	< 0.001
Succinate dehydrogenase (SDH)	172.47 ± 2.34	169.79 ± 12.64	174.73 ± 4.22	0.906
Malate dehydrogenase (MDH)	1598.75 ± 170.85	1559.86 ± 51.73	1565.42 ± 126.54	0.973
Lactate dehydrogenase (LDH)	11.09 ± 0.65^a^	3.82 ± 1.64^b^	6.03 ± 1.90^ab^	0.034

^a, b^ Means in the same row with different superscripts differed (*P* < 0.05)

### Histological analysis of the liver

**Fig. 1:**
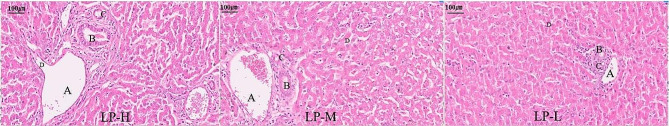
Liver tissue frozen sections. The ratio of Lys/Met is 1:1 in LP-L group, that Lys/Met ratio of LP-M group is 2:1, and that Lys/Met ratio of LP-H group is 3:1. HE staining, 20x. A: Interlobular veins; B: Interlobular bile ^duct;^ C: Interlobular artery; D: Hepatic Blood Sinusoids

As shown in Fig. 1, the hepatocytes of LP-L group displayed radial arrangement embracing the central vein, and the liver plates were tightly arranged. On the contrary, the hapatic lobules in LP-M group were separated from each other, accompanying by expansion of the blood sinuses. In LP-H group, the hepatocytes were loosely arranged, resulting in the separation of the liver plates from each other and a large perisinusoidal space.

### Characterization of liver tissue transcriptomic RNA-Seq data

A total of 521.73 million raw reads from nine liver samples were sequenced and mapped against the *Ovis* reference genome (Version 4.0). An average of 57.86 million clean reads were generated after quality control, with an average proportion of 99.79%. The Q20 values were ≥ 97.45% and Q30 values were ≥ 92.94%, in accordance with the requirement that Q20 should be over 95% and Q30 at least 85%. Approximately 94.80-98.35% of the clean reads were mapped to the *Ovis aries* reference genome, with single alignments in 32.86–44.41% of the reads **(**Table 5**)**.

**Table 5:** Sample sequencing data evaluation

Sample	Raw reads (M)	Clean reads (M)	Clean reads Q20	Clean reads Q30	Clean reads Ratio	Total mapping	Uniquely Mapping
LP-L-1	47.90	47.74	97.46%	92.99%	99.67%	95.73%	36.79%
LP-L-2	62.67	62.59	98.78%	94.93%	99.87%	97.92%	35.40%
LP-L-3	70.88	70.78	98.82%	95.15%	99.87%	97.58%	32.86%
LP-M-1	65.58	65.49	98.90%	95.51%	99.87%	98.11%	40.16%
LP-M-2	48.29	48.13	97.45%	92.94%	99.67%	96.56%	35.81%
LP-M-3	62.91	62.86	99.12%	96.8%	99.92%	98.35%	35.90%
LP-H-1	57.02	56.95	98.90%	95.42%	99.87%	97.96%	44.41%
LP-H-2	52.43	52.26	97.51%	93.12%	99.67%	96.27%	37.08%
LP-H-3	54.06	53.93	99.02%	96.39%	99.75%	94.80%	41.28%

### Analysis of DGEs

**Fig. 2:**
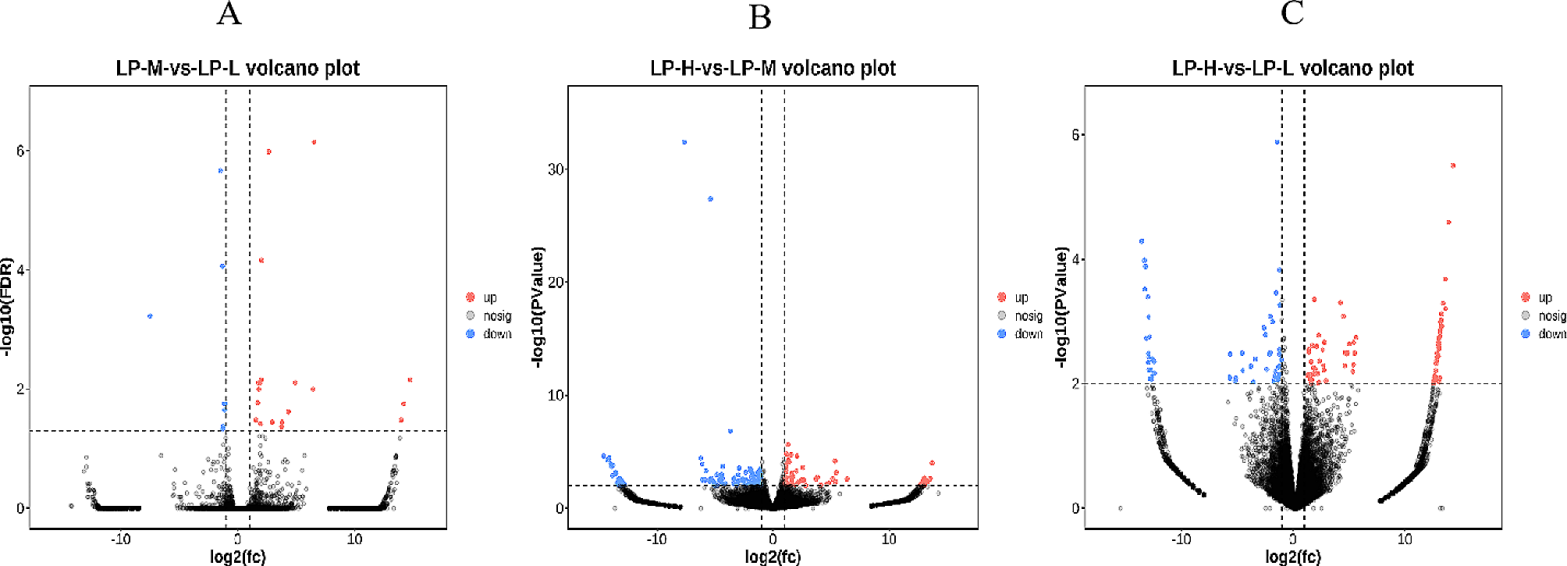
Volcano plot displaying DEGs within three different comparison groups. A: Compare the number of differential genes in liver tissue in the LP-M group and the LP-L group. B: Compare the number of differential genes in liver tissue in the LP-H group and the LP-M. C: Compare the number of differential genes in liver tissue in the LP-H group and the LP-L. up: Significantly upregulated genes; nosing: no significantly different genes; down: Significantly downregulated genes

A total of 337 DEGs were identified in three treatments. There were 21 upregulated and 8 downregulated genes in the LP-M group compared with the LP-L group. A comparison of the LP-M and LP-H groups indicated 179 DEGs, of which 66 were upregulated and 113 downregulated, while 129 DEGs were found between the LP-H and Lp-L groups, with 75 upregulated and 54 downregulated (Fig. 2).

### GO enrichment analysis

Gene Ontology (GO) is a standardized classification system for genes and includes three categories, namely, biological process (BP), cellular component (CC), and molecular function (MF). GO analysis of the DEGs showed that 242 biological process terms were significantly enriched in the LP-M group compared with the LP-L group, including activation of immune response, positive regulation of immune system process, activation of immune response, while a comparison of the LP-H and LP-M groups showed significant enrichment in 74 terms, including regulation of chemotaxis, immune response, and 52 terms were significantly enriched in the LP-H group compared with LP-L group, including regulation of extracellular exosome assembly, extracellular exosome assembly, TRAIL-activated apoptotic signaling pathway. The results showed DEG (*MYO9A*) involved in immune response was significantly upregulated in LP-L group.

**Fig. 3:**
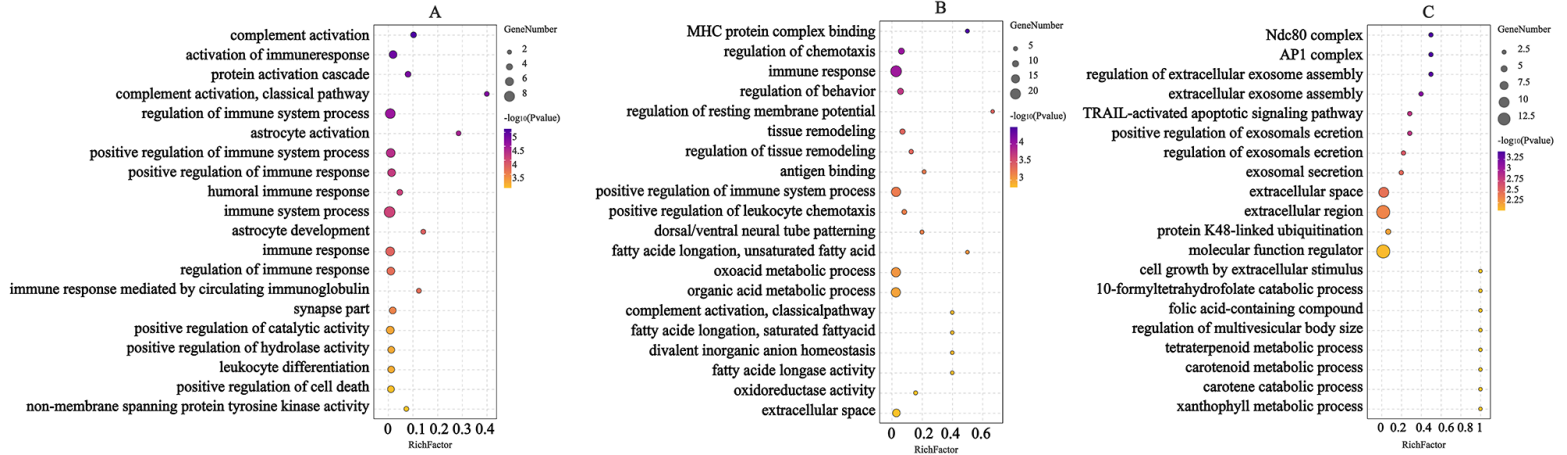
Top 20 GO entries within three different comparison groups. A: GO annotation of differently expressed genes between LP-M group and LP-L group; B: GO annotation of differently expressed genes between LP-H group and LP-M group; C: GO annotation of differently expressed genes between LP-H group and LP-L group. (X axis shows the rich factor of each GO term, color of the dot indicates the *p*-value, size of the dot denotes the number of genes involved in GO terms)

Moreover, the molecular function terms of non-membrane spanning protein tyrosine kinase activity, oxidoreductase activity, molecular function regulator were significantly enriched in the LP-M group compared with the LP-L group, LP-H group compared with the LP-M group and the LP-H group compared with the LP-L group respectively. Overall, the cellular components of synapse part, extracellular space, AP1 complex were significantly enriched in the LP-M group compared with the LP-L group, LP-H group compared with the LP-M group, the LP-H group compared with LP-L group respectively. The results indicated DEGs (*C1QA, JUNB*) involved in oxidoreductase activity, *PDK3* and *PDP2* involved in molecular function were significantly upregulated in LP-L group. (Fig. 3)

### PPI network analyses and correlation of phenotypic data with sequencing data

**Fig. 4:**
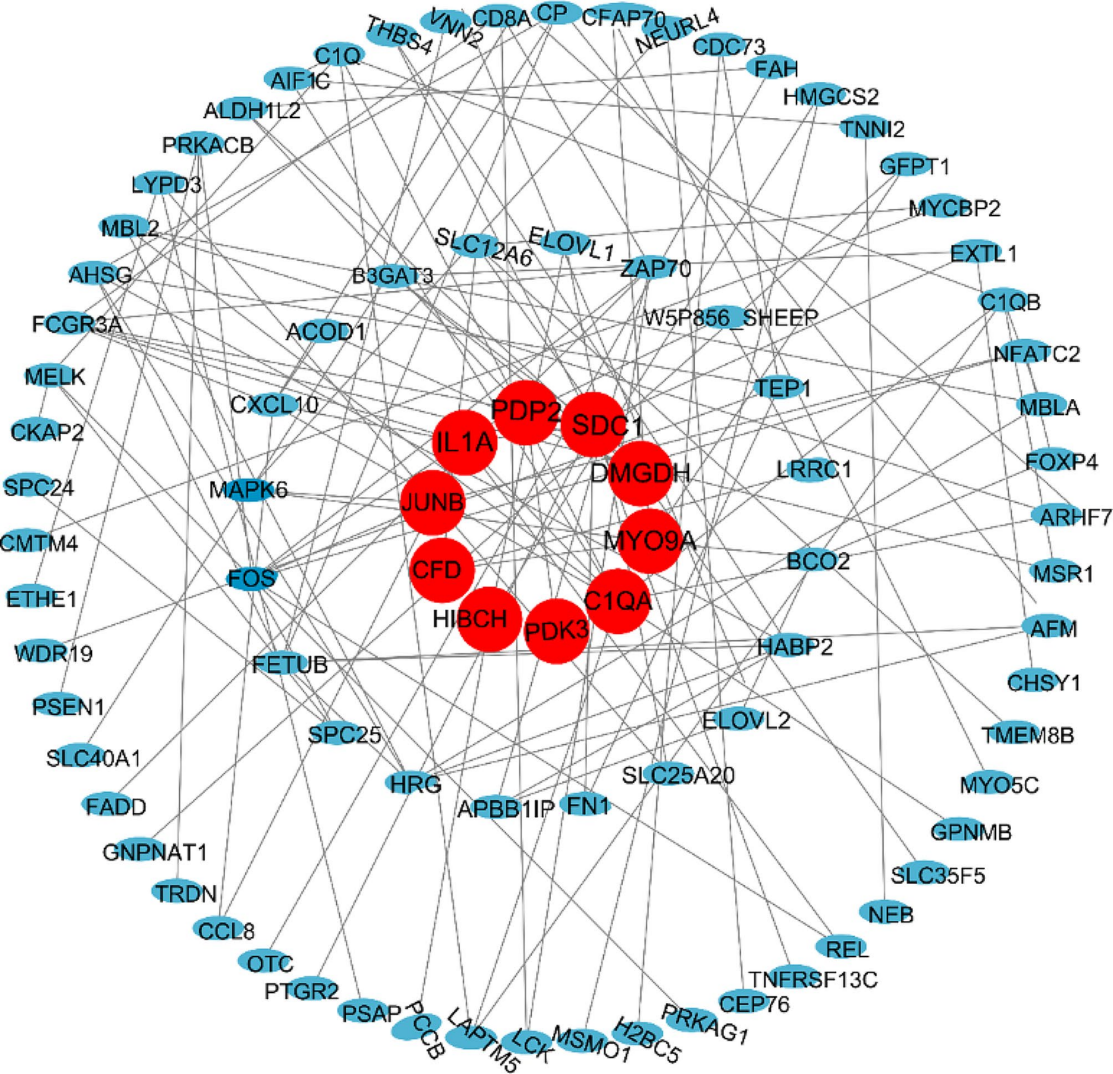
Protein-protein interaction (PPI) network. Red represents the core genes, Blue represents differentially expressed genes

**Fig. 5:**
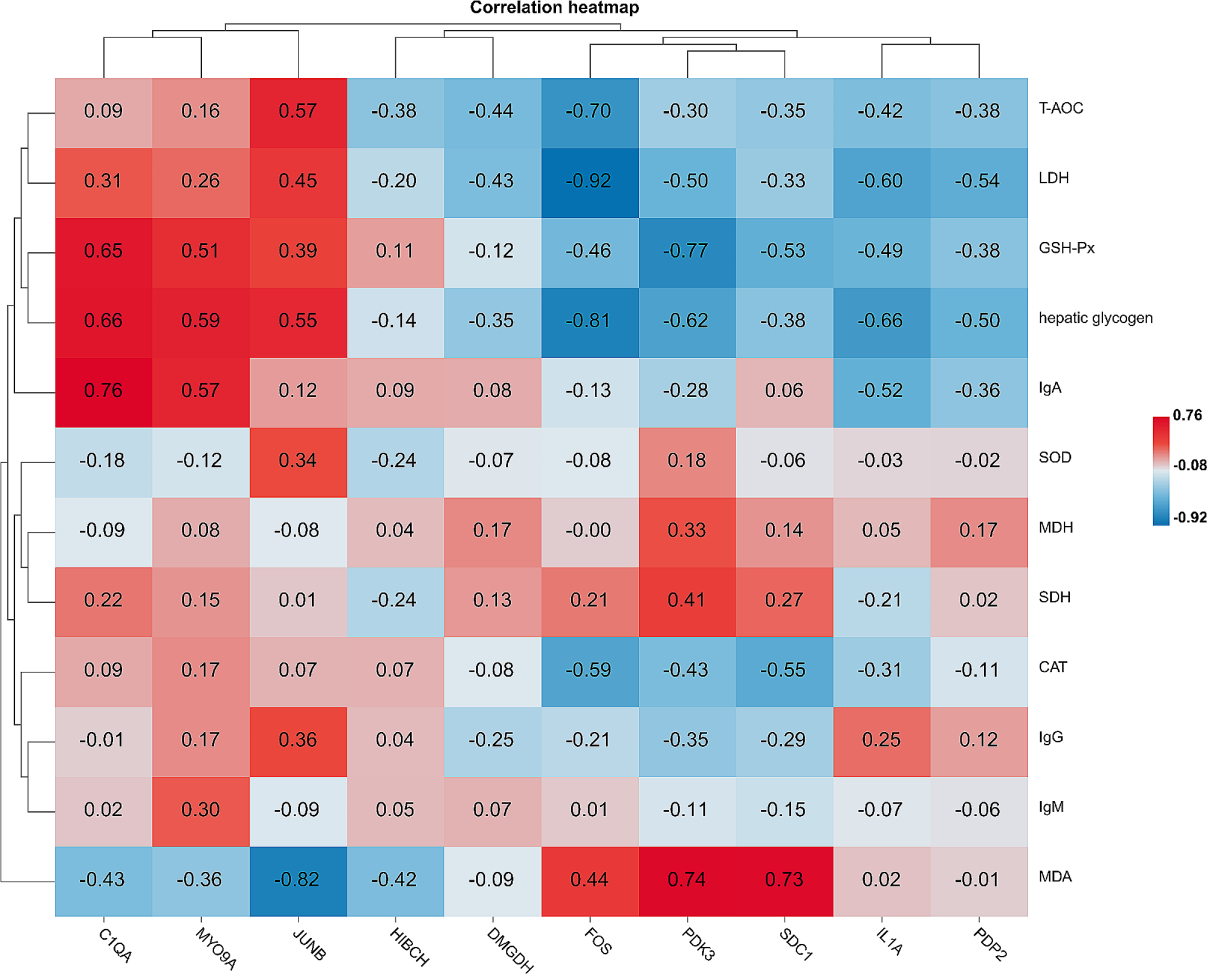
Correlation analysis of gene expression with liver antioxidant indexes and immune response and glycolytic enzyme activity indicators. Notes: R is Pearson correlation coefficient, and the closer R is to 1, the better the reproducibility of the sample building

PPI networks were constructed using the DEGs as seed nodes. The results showed that of the 337 DEGs, 10 genes interacted with one another other. These genes were fos proto-oncogene (*FOS*), myosin IXA (*MYO9A*), complement C1q A chain (*C1QA*), pyruvate dehydrogenase kinase 3 (*PDK3*), 3-hydroxyisobutyryl-CoA hydrolase (*HIBCH*), JunB proto-oncogene (*JUNB*), interleukin 1 alpha (*IL1A*), pyruvate dehydrogenase phosphatase catalytic subunit 2 (*PDP2*), syndecan 1 (*SDC1*), and dimethylglycine dehydrogenase (*DMGDH*) (Fig. 4). In addition, the Pearson correlation coefficient was used to investigate the potential associations between the expression levels of these genes and antioxidant capacity, immune response, and glycolytic activity were also investigated. The results showed that *C1QA* and *JUNB* were positively correlated with CAT, T-AOC, and GSH-Px and negatively correlated with MDA. *MYO9A* was positively correlated with IgA, IgG, and IgM levels **(**Fig. 5**).**

### Verification by qRT-PCR

**Fig. 6:**
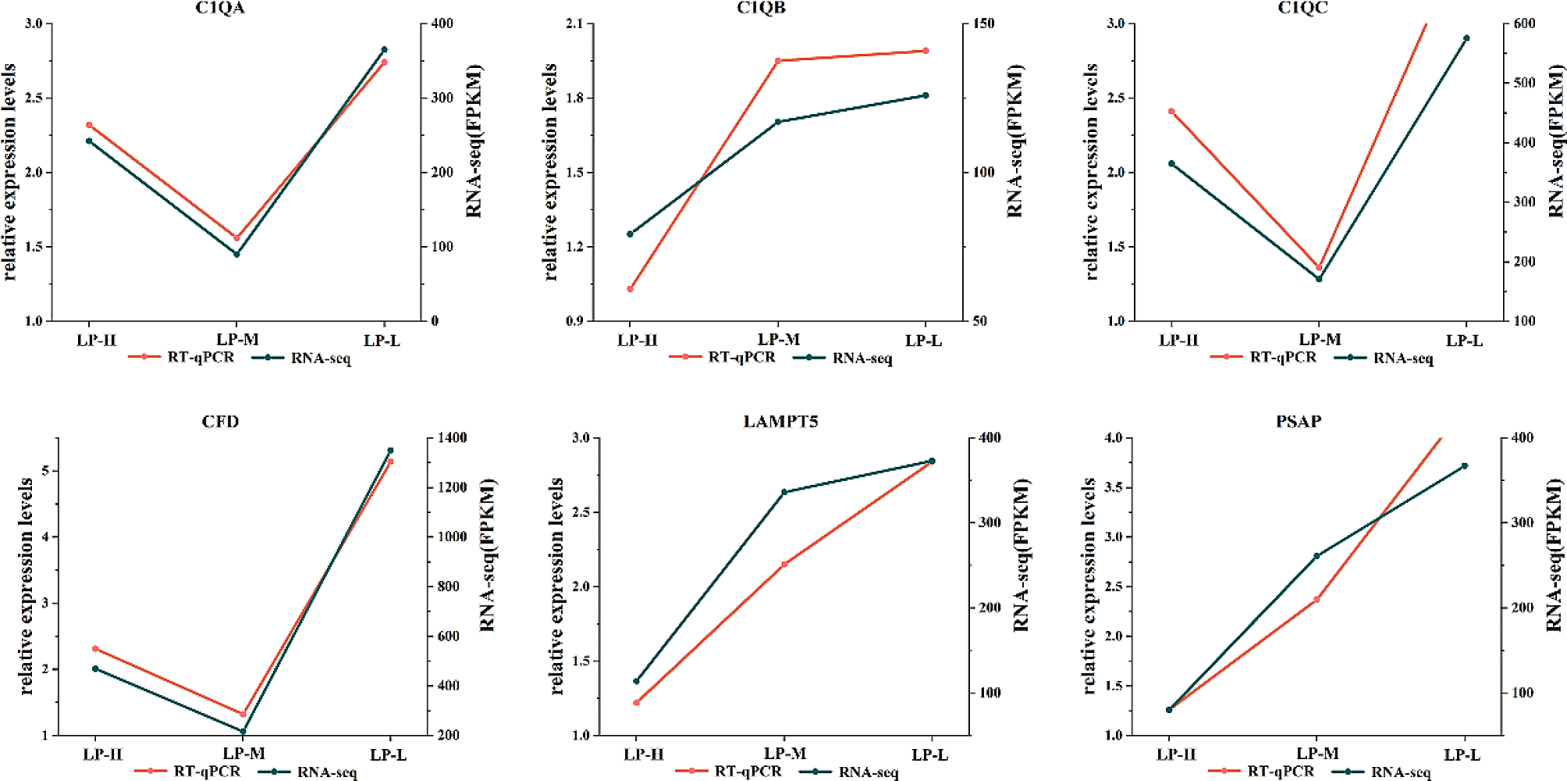
The figure shows the quantitative results of RNA-seq and qRT-PCR; the blue line shows the FPKM value of the sequencing and the pink line shows the relative quantitative results

To verify the accuracy of the RNA-Seq results, six DEGs that were associated with antioxidant activity, immune function, and glycolytic enzyme activity were selected for qRT PCR quantification. It was found that the expression trends in both the qRT-PCR and RNA-Seq results were consistent, indicating the reliability of the RNA-Seq results **(**Fig. 6**)**.

## Discussion

In ruminant, meeting AA requirements becomes more crucial during stages of high growth performance. Previously, Hanwoo Steer supplemented with Met had improved average daily gain and a lower feed conversion ratio compared to control in high-temperature seasons [16]. Similarly, the average daily gain was increased, while feed to gain ratio was decreased with the increasing Met supplementation in feedlot Yaks [17]. In the present study, the final body weight with Lys/Met at ratio 1:1 was increased of Tibetan sheep. Our outcomes were consistent with previous studies, which provided evidence that dietary Met improved the final body weight and carcass weight by altering rumen fermentation in Hu sheep [18]. One possible explanation is that, dietary Met supplementation improved the feed efficiency and nitrogen utilization, thereby contributing to the growth performance [19].

Many studies have explored more effective use of Lys and Met in diets, not just to meet the nutritional needs but also as a health product. In Pekin ducks, dietary Met deficiency affected histological characteristics of the liver via suppression of fatty acids transportation and hepatic catabolism [20]. Meanwhile, the liver injury including swollen, blunt, turbid and lusterless were observed morphologically when fed Met-deficient diet in broilers, which might be related to oxidative stress and intestinal immunosuppression [21]. Our results showed that increasing the dietary Met/Lys ratios contributed to improvement of hepatic phenotype in Tibetan sheep. Previous study showed that the lipopolysaccharides (LPS) induced liver injury, while dietary Met supplementation alleviated these damages [22]. Therefore, we speculated that high ratio of Met/Lys in diet resulted in decreases of LPS concentration in the liver, which alleviated the LPS-induced negative effects.

Oxidative stress results from an unbalance between antioxidants and oxidative molecules, which is a vital contributing factors to the impairment of the immune responses during environmental stress [23]. According to previous studies, responses of oxidative stability to supplemental Lys or Met were variable in livestock. Zhan et al. (2006) suggested that higher hepatic concentration of carnitine, synthesised from Met and Lys, could facilitate fatty acid oxidation in the liver [24]. Liang et al. (2019) found that adding N-acetyl-l-methionin to a diet reduces lipid peroxidation and increases hepatic protein synthesis in in mid-lactating dairy cows [25]. Among the antioxidant indexes, both detoxification of free radicals and non-radical harmful chemicals (i.e., GSH-Px, SOD, CAT, T-AOC) constitute the antioxidant defense strategy [19]. Previous study indicated that high stocking density resulted in decrease of T-AOC and GSH-Px activities, and that effect was mitigated by dietary Met supplementation [26]. In this study, the GSH-Px and CAT activities was obviously increased with increasing dietary Met/Lys ratios, which is in agreement with previous findings. MDA is a lipid peroxide and its overproduction destroys the integrity of cell membranes and promotes mutation, thereby reducing the effectiveness of the oxidative defense system [27]. Our results showed that the MDA concentration in the liver significantly decreased with an increase in the Lys/Met ratios. In accordance with our findings, MDA concentration in the small intestine were affected by Met levels, high dietary Met supplement significantly reduce MDA concentration [28]. Overall, increasing the dietary Met/Lys ratios contributed to enhancement of hepatic oxidative stability in Tibetan sheep. The main reason may be that the exogenous Met could increase eliminated reactive oxygen species by Met residues or through glutathione synthesis, directly and indirectly, influence the activity of the antioxidant enzymes [29].

The immunoglobulins were secreted by B lymphocytes and played play a critical role in adaptive immune system [30]. Among the five Igs, IgA was involved in antisepsis, sterilization, and antivirus [31]. IgG is an important immunoglobulin in the serum, accounting for approximately 75% of the total immunoglobulin content in the serum. contributing to the modulation of immunity [32]. Gebeyew et al. (2021) showed that the plasma IgA concentration increased when supplementing Met and Lys in dairy sheep [12]. Similarly, dietary supplementation of Zn-methionine increased the blood IgG concentration and decreased rumen ammonia-N compared to the control diet [33]. Although no significant difference, increasing the dietary Met/Lys ratios tended to increase the concentration of IgA and IgG in the liver of Tibetan sheep. This is consistent with the findings of Zhou et al. (2017), who observed that during the transition period, when dietary Met was fed, liver function and inflammation improved in dairy cow [34]. One possible explanation is that, dietary Met supplementation modulated the expression of immunity-related genes including *TNNT3*, *PALLD* and *PYGM* by altered alternative splicing and DNA methylation [35], thereby altering the immune status of the liver.

Glycolysis converts glucose into pyruvate, and synchronously provides 2 reduced nicotinamide adenine dinucleotides (NADH) and 2 adenosine triphosphates (ATP), which meets the cellular energy required for diverse biological activities [36]. Glycogen is synthesized mainly by several enzymes such as glycogen synthase (GS) and glycogen synthase kinase 3β (GSK-3β) [37] and participate in maintenance of energy homeostasis [38]. Mammalian LDH is a ubiquitous intracellular enzyme that catalyses pyruvic acid to lactate via the Cori cycle [39], which delays ongoing metabolic acidosis [40] and liver injury [41]. In the current experiment, high Met supplementation increased the activity of enzymes (glycogen and LDH) involved in the hepatic glycolysis in Tibetan sheep. Previously, expression of key proteins related to fatty acid transport, fatty acid oxidation and glycolysis/gluconeogenesis were suppressed when dietary Met deficiency, which were ameliorated by feeding diets with adequate Met [20]. Compared with low dietary Met, high Met supplement enhanced the mRNA levels of hepatic gluconeogenesis related genes such as phosphoenolpyruvate carboxykinase and glucose-6-phosphatase [42], which was effective in improving energy metabolisms in late pregnant ewes [43].

At least in part, gene expression levels might parallel well with its corresponding function in mammals [44]. Dietary Met supplementation in sheep during late pregnancy improved the offspring development with a concomitant change in hepatic markers involved in the energy metabolism (i.e., *FABPF*, *AlOX15* and *COX-2*) [45]. A similar result was found in sheep which were fed the Met diet increased the improve ewe performance and fetus development via modulating the expression of *AHCY* and *ALOX5P* of the liver in sheep [46]. In the Holstein cows, Met supplementation in dite altered the expression of *FOXO1*, *PPARG*, *E2F1*, and *CREB1*, thereby elevating the metabolic stress in newborn liver [47]. In the current study, 337 genes were found to be differentially expressed in the liver fed different proportion of Lys/Met diets of Tibetan sheep.

Six of these DEGs, including *C1QA*, *C1QB*, *C1QC*, *CFD*, *LAPTM5*, and *PASP* were identified as candidate genes that might be involved in regulating hepatic oxidative stress, immune response and glycolysis. As a serine protease, the *CFD* is predominantly produced by adipocytes, which play an essential role in the activation of the alternative pathway of the complement system [48]. *C1QA*, *C1QB*, and *C1QC* belong to the family of complement C1Q and are involved in immune response [49]. MHV A59-infected *C1QA* knockout mice exhibited significantly hepatocellular necrosis and interstitial pneumonia compare with wild-type mice, resulting in immune deficiency [50]. *C1QB* regulated the immune response by altering the number of macrophages and T lymphocytes in the pancreatic islet in rats [51]. *C1QC* is a polypeptide involved in immune system-related biological processes via closely linking with immune-related signaling pathways (i.e., leukocyte activation, leukocyte activation and inflammatory response) [52]. *PASP* encoded for the lysosomal protein prosaposin and closely related with mTOR signaling. In *vitro*, the silencing of *PASP* suppressed glycolysis and oxidative phosphorylation [53]. Currently, our analysis revealed consistent correlations between the RNA-Seq results and the mRNA levels measured by qRT-PCR. We speculated that low-protein diet with a low Lys/Met ratio (1:1) could significantly reduce the hepatic oxidative stress and improve the glycolytic activity of Tibetan sheep by modulating the expression of related functional genes.

## Conclusions

In summary, feeding low dietary protein (10%) with a low Lys/Met ratio (1:1) to Tibetan sheep could improved the body weight and hepatic phenotype, as well as modulation of genes expression associated with antioxidant capacity, immune function, and glycolytic activity in liver. Our findings indicate that providing a low protein diet supplemented with optimal Lys/Met ratio (1:1) could benefit mainly by reducing the hepatic oxidative stress and improving the glycolytic activity.

## Data Availability

The data sets utilized in this article are available on request from the author(1960742393@qq.com). The data presented in this study are openly available in Sequence Read Archive at https://www.ncbi.nlm.nih.gov/sra (accessed on 2 March 2024), reference number PRJNA1081789 and PRJNA1073180.
